# Early innate immune responses in different COVID‐19 sub‐phenotypes through a transcriptomics lens

**DOI:** 10.1002/ctd2.79

**Published:** 2022-06-01

**Authors:** Wei Yang

**Affiliations:** ^1^ Departments of Surgery and Biomedical Sciences Cedars‐Sinai Medical Center Los Angeles California USA

The coronavirus disease 2019 (COVID‐19) pandemic, caused by the severe acute respiratory syndrome coronavirus 2 (SAR‐CoV‐2), has caused over 520 million infections and over 6 million deaths worldwide since December 2019 (https://covid19.who.int/, accessed on May 14, 2022). COVID‐19 causes a wide range of symptoms and severity levels.^1^ Although most cases (∼80%) are asymptomatic or have mild to moderate symptoms, ∼15% develop severe illness, ∼5% progress to critical illness, and ∼2% are fatal.^2^

This wide spectrum of disease severity can be attributed to the virus itself, the environment, and the host.^3^ The SARS‐CoV‐2 virus is constantly evolving into new variants. Among these, five variants have been designated by the World Health Organization as variants of concern: alpha, beta, gamma, delta, and omicron. These variants are associated with varying degrees of disease severity; for example, the delta variant is associated with more severe disease than the alpha and omicron variants in unvaccinated populations.^4^ COVID‐19 susceptibility and severity are also associated with environmental factors such as air pollution, chemical exposures, climate, and the built environment.^5^ Nonetheless, it appears that the host is the most important factor in explaining disease heterogeneity, infection rates, and long‐term medical consequences.^6^

A number of host‐related factors such as age, gender, race, and comorbidities are significantly associated with COVID‐19 severity.^7^ These risk factors, however, do not fully explain the variation in disease severity between individuals. Innate immunity, our immune system's first line of defense, is critical in the fight against viruses. Variability in innate immune system components among individuals is believed to be a major contributor to the diverse disease courses observed for COVID‐19. Nonetheless, little is known about the COVID‐19 severity‐related early molecular changes in the host immune response to SARS‐CoV‐2 infection.

In a study published in *Clinical and Translational Medicine*, Maurya et al. reported unique early innate immune responses in different sub‐phenotypes of COVID‐19, through transcriptomic analysis of nasopharyngeal or throat swab samples collected on the day of hospital admission (Figure 1). Among a total of 125 cases, 107 were recovered from COVID‐19 and 18 died as a result of it. Transcriptomic analysis revealed that genes upregulated in the recovered group are primarily involved in epithelial integrity and mucosal immunity, indicating an active defense against SARS‐CoV‐2 infection. Among the 107 recovered cases, 62, 31, and 14 had mild, moderate, and severe symptoms, respectively. A more detailed pairwise comparison of the mild, moderate, severe, and mortality groups revealed that the S100A2, TLR, LRP, and IL1R2 pathways may be important for disease severity. Because the need for respiratory support (RS) is indicative of disease severity, the authors compared the transcriptomic profiles of three RS subgroups: no‐RS, RS, and ventilator support (VS). Several immune response genes related to COVID‐19 and other infectious diseases were discovered to be upregulated in the VS group, implying an active host immune response. Furthermore, network modelling suggested that activation of the alternative complement and JAK‐STAT pathways are potential mechanisms underlying the need for VS.

**FIGURE 1:**
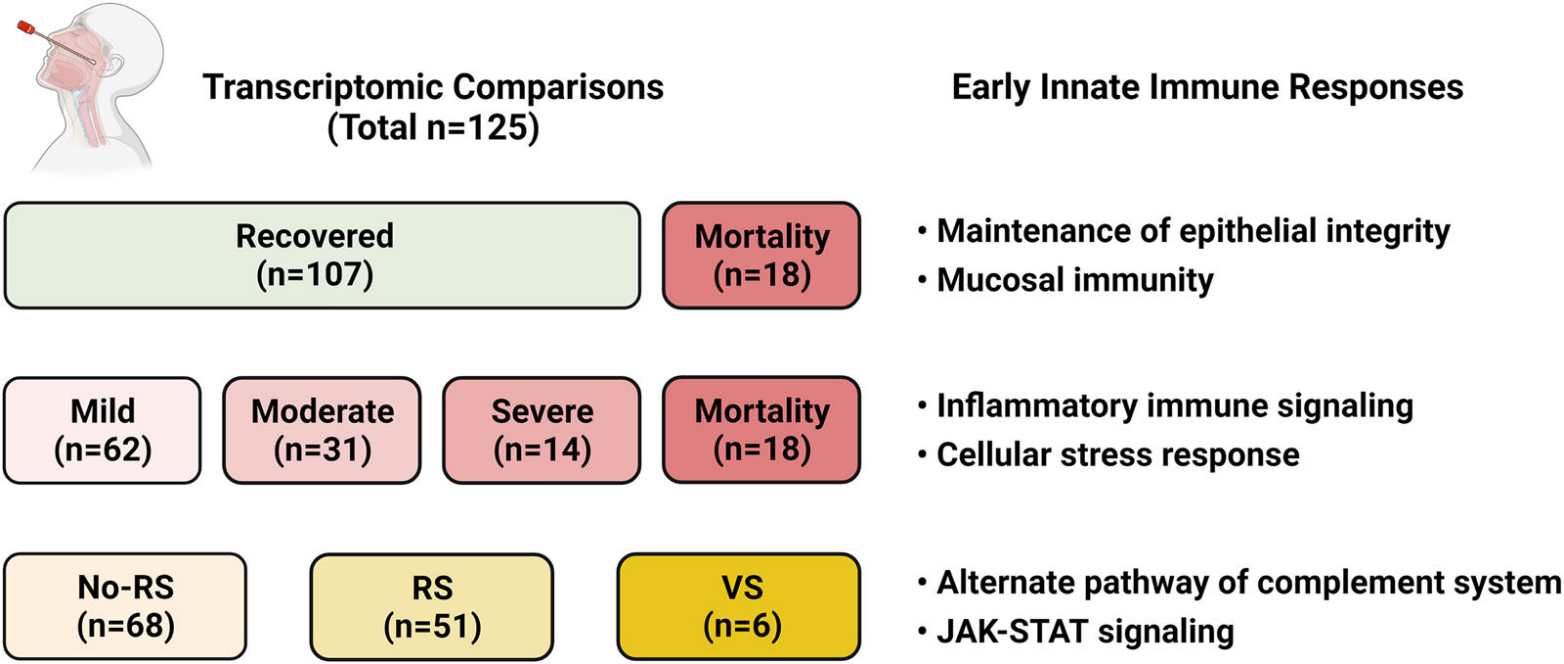
Diagrammatic summary of the Maurya et al. study. RS: respiratory support; VS: ventilator support. Figure created using biorender.com

Beyond providing new insights into COVID‐19 severity, the differentially expressed genes identified in this study can be further developed into candidate gene signatures to predict disease severity and the need for RS. If validated in larger and independent patient cohorts, these prognostic biomarkers will significantly improve clinical decision‐making and thus patient outcomes.

In addition, the strategy described in this study can be used to help understand the molecular mechanisms underlying persistent COVID, also known as long‐haul COVID or long COVID. It was estimated that about 10% of COVID‐19 survivors are long‐haulers who suffer from ongoing symptoms for an extended period of time.^8^ However, the underlying molecular mechanisms remain poorly understood.^9^ Multi‐omics analysis of clinical specimens from COVID‐19 patients with and without long‐term symptoms will help us understand the underlying molecular mechanisms, discover novel diagnostic and prognostic biomarkers, and identify clinically actionable drug targets to combat this new disorder that affects millions of people worldwide.

## CONFLICT OF INTEREST

The authors declare that there is no conflict of interest that could be perceived as prejudicing the impartiality of the research reported.

## References

[1] Vetter P , Vu DL , L'Huillier AG , Schibler M , Kaiser L , Jacquerioz F . Clinical features of covid‐19. BMJ. 2020;369:m1470. 32303495 10.1136/bmj.m1470

[2] Papadopoulou G , Manoloudi E , Repousi N , Skoura L , Hurst T , Karamitros T . Molecular and clinical prognostic biomarkers of COVID‐19 severity and persistence. Pathogens. 2022;11(3):311. 35335635 10.3390/pathogens11030311 PMC8948624

[3] Morens DM , Fauci AS . Emerging pandemic diseases: how we got to COVID‐19. Cell. 2020;182(5):1077‐1092. 32846157 10.1016/j.cell.2020.08.021 PMC7428724

[4] Lauring AS , Tenforde MW , Chappell JD , et al. Clinical severity of, and effectiveness of mRNA vaccines against, covid‐19 from omicron, delta, and alpha SARS‐CoV‐2 variants in the United States: Prospective observational study. BMJ. 2022;376:e069761. 35264324 10.1136/bmj-2021-069761 PMC8905308

[5] Weaver AK , Head JR , Gould CF , Carlton EJ , Remais JV . Environmental factors influencing COVID‐19 incidence and severity. Annu Rev Public Health. 2022;43(1):271‐291. 34982587 10.1146/annurev-publhealth-052120-101420 PMC10044492

[6] Schultze JL , Aschenbrenner AC . COVID‐19 and the human innate immune system. Cell. 2021;184(7):1671‐1692. 33743212 10.1016/j.cell.2021.02.029 PMC7885626

[7] Williamson EJ , Walker AJ , Bhaskaran K , et al. Factors associated with COVID‐19‐related death using OpenSAFELY. Nature. 2020;584(7821):430‐436. 32640463 10.1038/s41586-020-2521-4 PMC7611074

[8] Greenhalgh T , Knight M , A'Court C , Buxton M , Husain L . Management of post‐acute covid‐19 in primary care. BMJ. 2020;370:m3026. 32784198 10.1136/bmj.m3026

[9] Mehandru S , Merad M . Pathological sequelae of long‐haul COVID. Nat Immunol. 2022;23(2):194‐202. 35105985 10.1038/s41590-021-01104-y PMC9127978

